# Does Emotional Intelligence have a “Dark” Side? A Review of the Literature

**DOI:** 10.3389/fpsyg.2016.01316

**Published:** 2016-08-30

**Authors:** Sarah K. Davis, Rachel Nichols

**Affiliations:** Psychological Sciences, Institute of Health and Society, University of WorcesterWorcester, UK

**Keywords:** emotional intelligence, dark side, psychological health, stress reactivity, emotional manipulation, deception, dark triad, antisocial behavior

## Abstract

Emotional intelligence (EI) was once touted as the panacea for a satisfying and successful life. Consequently, there has been much emphasis on developing interventions to promote this personal resource in applied settings. Despite this, a growing body of research has begun to identify particular contexts when EI does not appear helpful and may even be deleterious to a person, or those they have contact with, suggesting a “dark” side to the construct. This paper provides a review of emergent literature to examine when, why and how trait and ability EI may contribute to negative intrapersonal (psychological ill-health; stress reactivity) and interpersonal outcomes (emotional manipulation; antisocial behavior). Negative effects were found to operate across multiple contexts (health, academic, occupational) however these were often indirect, suggesting that outcomes depend on pre-existing qualities of the person. Literature also points to the possibility of “optimal” levels of EI—both within and across EI constructs. Uneven profiles of self-perceptions (trait facets) or actual emotional skills contribute to poorer outcomes, particularly emotional awareness, and management. Moreover, individuals who possess high levels of skill but have lower self-perceptions of their abilities fare worse that those with more balanced profiles. Future research must now improve methodological and statistical practices to better capture EI in context and the negative corollary associated with high levels.

## Introduction

Emotional intelligence (EI) is broadly defined as competency in perceiving, understanding and regulating our own emotions and the emotions of others (Zeidner et al., 2009). Two perspectives permeate the literature, *trait* EI (TEI: emotion relevant self-perceptions and dispositions e.g., empathy, self-control) and *ability* EI (AEI: cognitive abilities specialized for emotional information processing e.g., emotion perception, understanding), which are distinct in their measurement methods and underlying empirical bases (Petrides et al., 2007; Mayer et al., 2008). It was once argued that high levels of EI could be more beneficial for success than known predictors of performance, particularly intelligence (Goleman, 1995). Myriad Social and Emotional Learning programmes (SEL) and adult training packages were consequently developed and implemented in educational (e.g., Brackett et al., 2012) and workplace settings (e.g., Grant, 2007), with the goal of enhancing these emotional self-perceptions and skills for successful adjustment.

Over the past two decades, research has supported a link between EI and adaptive life outcomes, including better mental and physical health (Martins et al., 2010), academic (Perera and DiGiacomo, 2013), and occupational success (Joseph and Newman, 2010). Nevertheless, with statistical control for allied performance factors (e.g., personality; IQ), many of these effects are not of the original size and strength predicted by early EI proponents (Matthews et al., 2012). Moreover, whilst evaluations of SEL training programmes have revealed benefits for mental health, pro-social behavior and academic achievement, effects are typically moderate in size and of questionable longevity (Durlak et al., 2011). Thus, whilst EI appears to contribute some adaptive value to life outcomes, the original fervor for the construct has been tempered.

Simultaneously, a growing body of research has begun to identify particular contexts when EI does not appear helpful, and even deleterious to a person, or those they have contact with, suggesting there is a “dark” side to the construct. For instance, high levels of AEI conferred vulnerability for internalizing symptoms in adults facing chronic stress (Ciarrochi et al., 2002), whilst in occupational settings, EI was used as a tool for emotional manipulation of others (Côté et al., 2011). The concept of dark traits and abilities is not new. There is a rich literature examining prototypically negative personality traits—Machiavellianism; Psychopathy; Narcissism which are characterized by callous and manipulative behaviors and frequently linked to nefarious antisocial outcomes (Furnham et al., 2013). Indeed, some research has reported positive associations between high TEI and Narcissism (e.g., Petrides et al., 2011; Zhang et al., 2015), indicating that similar antisocial outcomes may befit high TEI scorers. Grant and Schwartz (2011) postulate even prototypically positive skills and virtues, e.g., loyalty and empathy, can have decreasing returns at increasing levels—at very high levels, any personal benefits are outweighed by a negative impact on adjustment (“nonmonotonic” effects). This is well-documented in the case of *self-esteem* which has been associated with better academic, psychological, and social adjustment (Zeigler-Hill et al., 2016), yet there is ample evidence that highly inflated (and unstable) levels of self-esteem can have negative consequences, including aggression (for review see Baumeister et al., 2003). A similar pattern has been documented for *self-efficacy*—confidence in one's ability to meet challenges and achieve goals (Ehrlinger and Eichenbaum, 2016). For instance, self-effacing (under-confidence in cognitive abilities) and self-enhancing (over-confidence in abilities) beliefs resulted in increased depression and higher levels of dejection-related emotion in students following inconsistent performance feedback (Kim and Chiu, 2011). This provides support for the idea of an “optimal” level for such positive illusions (e.g., Baumeister, 1989).

There is therefore theoretical and empirical support for dark EI. As a multifaceted construct embodying skills (AEI), self-perceptions, and dispositions (TEI) relating to self and others (e.g., assertiveness; emotion management), very high levels of EI could convey negative internal or “intrapersonal” effects for the person concerned, but also negative external or “interpersonal” effects involving others. Following a timely review of the literature, this paper seeks to explore these possibilities further by addressing the following questions: *When (in which contexts), why (which facets), and how (directly or indirectly) might EI be deleterious or harmful? Is there an optimal level of EI?* Hence, this review aims to synthesize a growing number of findings which do not fit with the dominant empirical position that higher EI is always better.

English language empirical studies, published in peer-reviewed journals since 1990, were retrieved via PsycInfo and ScienceDirect. Keywords included *emotional intelligence, negative effect, deleterious, damaging, harmful, disadvantage*. Only studies using validated measures of T/AEI, derived from established models of EI (see Table 1 for exemplars) and those that reported statistically significant relationships between EI and outcome variables, were included. After removing duplicates, reviewing titles, and abstracts, 38 studies were retained from an initial pool of 308. 24 of these met all above-mentioned inclusion criteria upon reading in full. The 14 studies excluded at this stage alluded to but did not report statistically significant detrimental effects related to EI (e.g., Balluerka et al., 2013). A further 10 articles were added from a manual search of the reference list of each study retrieved, producing a final set of 34 articles. Studies were coded for reference information, methodological details (EI tool/sample demographics), and key findings. Outcome variables were classified as being either “intrapersonal” (e.g., mental health status) or “interpersonal” (e.g., deviant behavior) in nature (see Table 1).

**Table 1:** **Intrapersonally and interpersonally “dark” effects associated with high emotional intelligence**.

**Study**	**EI tool**	**Outcome variable(s)**	**Sample**	**Summary of results**
**INTRAPERSONAL EFFECTS**				
Bechtoldt and Schneider, 2016	MSCEIT (German)	Cortisol reactivity	*N* = 166 (all male). *M* = 21.2, *SD* = 3.2.	EI associated with higher acute stress in a socially demanding context, although basal testosterone levels moderated this. High EI individuals also had slower recovery from heightened cortisol.
Ciarrochi et al., 2002	MEIS (emotion perception); SSEIT	Stress (daily hassles; major life events); mental health	*N* = 302 (232 female) *M* = 20.6, *SD* = 5.0	Higher emotion perception associated with greater depression, suicidal ideation, and hopelessness in individuals reporting high levels of daily hassles (though not life events). Higher self-reported competency in managing the emotions of others was related to less suicidal ideation in those experiencing high levels of daily hassles.
Davis and Humphrey, 2012a	TEIQue-ASF, MSCEIT-YV R	Depression; disruptive behavior	*N* = 412 (214 female). *M* = 13.09, *SD* = 1.07	AEI amplified relationship between economic deprivation and depression. TEI attenuated relationship between family dysfunction and disruptive behavior.
Davis and Humphrey, 2014	TEIQue-ASF, MSCEIT-YV R	Psychological adaptation	*N* = 1170 (558 female). *M* = 13.03, *SD* = 1.26.	Average to high AEI coupled with low TEI produce poorer results i.e., greater depression. This effect is most pronounced in those with AEI in top 10% and TEI in bottom 10%, and particularly in the conditions of family dysfunction and socioeconomic adversity.
Elipe et al., 2015	TMMS (Spanish)	Cyber victimization	*N* = 636 (68.7% female). *M* = 20.45, *SD* = 4.13	EI acts as a moderator variable between cyber victimization and negative emotional impact (annoyance and dejection). Clarity, however, has positive relationship with negative impact, particularly with low levels of Repair.
Extremera and Fernandez-Berrocal, 2006	TMMS (Spanish)	Psychological wellbeing	*N* = 169 (136 female). *M* = 22.84, *SD* = 4.24.	Emotional Attention correlated positively with depression and anxiety, and negatively with dimensions of Role Emotional, Social Functional, and Mental Health, unlike Clarity and Repair.
Fernández-Berrocal and Extremera, 2006	TMMS	Affect sensitivity after mood induction	*N* = 155 (123 women). *M* = 22, *SD* = 2.66	Global negative affect increased in response to a film clip selected to induce anger for those high in clarity. However, changes in positive affect following positive induction did not differ according to level of TEI. High clarity poorer mood (more negative affect) at time 3 (recovery phase). Those high in clarity and low in repair took the longest to recover (mood state) post-stressor.
Gohm et al., 2005	MSCEIT and TMMS	Stress	*N* = 158 (97 female, 13 unreported). *M* = 18.3	High levels of AEI differentially related to lower levels of perceived stress as a function of TEI (“Attention,” “Clarity,” “Intensity”); AEI appeared advantageous when “clarity” and “intensity” were either uniformly high or low, but was not beneficial for those who were potentially most vulnerable—individuals experiencing intense emotions but who have a lack of perceived emotional understanding (clarity), termed “overwhelmed.”
Keefer et al., 2012	EQ-i:S	Degree completion	*N* = 1015 students (732 female, 1 unreported). *M* = 19.23, *SD* = 0.71	Successful degree completion less likely with low-EI profile but, counter-intuitively, not more likely for those with a high-EI profile. Having skill in at least one area important but low-average interpersonal/intrapersonal skills and adaptability coupled with *high* stress management yielded higher rates of degree non-completion.
Li et al., 2015	SSEIT (Chinese)	Post-traumatic growth	*N* = 260 (all female). *M* = 19.4, *SD* = 0.8	Nursing students with a history of childhood adversity with low or high EI reported lower levels of growth than those with average EI.
Lizeretti et al., 2012	TMMS (Spanish)	Clinical symptoms in mental disorders	*N* = 326 (50% met clinical diagnoses, 50% control)	All clinical groups (particularly anxiety disorder and borderline personality disorder) scored higher in Attention to Feelings than control group. Clarity was the most significant factor in distinguishing between clinical/non-clinical groups: lack of clarity could lead to mistrust of emotions and avoidant coping techniques.
Lyons and Schneider, 2005	MSCEIT	Academic task performance	*N* = 126 (60% female). *M* = 20, *SD* = 4.6	After controlling for GPA, higher EI (perceiving, facilitating thoughts) related to better performance in a speech delivery task with emotive content in females. However, related to poorer performance in males—may have been uncomfortably aware of emotive content.
Matthews et al., 2006	MSCEIT	Task-induced stress	*N* = 200 (132 female). *M* = 19.7, *SD* = 3.1	High EI individuals experienced greater post-task stress.
Petrides and Furnham, 2003	EQ-I TEIQue	Emotion recognition and affect sensitivity after mood induction	Study 1: *N* = 34 (25 females). *M* = 20.27, *SD* = 1.23	Study 1: High TEI related to faster emotion perception (happy faces recognized fastest of all 5 emotions)
			Study 2: *N* = 30 (22 females). *M* = 20.69, *SD* = 2.95	Study 2: Higher TEI reported more anxiety, anger and reduced vigor after watching a distressing film (and reduced confusion following an amusing film) compared to those with lower TEI.
Rego et al., 2010	Rego et al. (2010) EI scale	Caring behaviors in nurses	Nurses: *N* = 120 (75% female). *M* = 32. Patients: *N* = 360 (43% female). *M* = 49.6.	Nurses high on empathy had fewer caring behaviors if they were also high on emotional self-control. Nurses overall scored relatively low on emotional self-control. Question over whether self-control actually prevents emotional displays that may aggravate situation.
Salguero et al., 2015	MSCEIT SSEIT	Depression	*N* = 213 (all female). *M* = 20.12, *SD* = 5.45.	TEI strongly and negatively correlated with depression, but no such relationship for AEI. TEI and AEI only moderately related (*r* = 0.30). TEI moderated the relationship between AEI and depression such that a reduction in depressive symptoms was only found in those with high TEI as well as high AEI. Women who had poorer emotional skills and those who believed they did had greater levels of depression.
Sevdalis et al., 2007	TEIQue-SF	Affect sensitivity under mood induction	Study 1: *N* = 60 (43 female, 1 unreported). *M* = 25.24, *SD* = 9.69. Study 2: *N* = 24 (14 female). *M* = 22.21, *SD* = 2.81.	Study 1: Positive affect decreased and negative affect increased more in individuals with high TEI after recalling a regretful decision, although significantly different baseline PA and NA not taken into consideration. Study 2: Individuals with high TEI over predicted the amount of regret felt five days after a failed negotiation, and generally felt better.
Thayer et al., 2003	TMMS	Depressive Symptoms	*N* = 175 (99 female). *M* = 20.9.	Although men and women did not differ significantly on Clarity or Repair, women scored higher on Attention. This difference in Attention accounted for 14% variance in gender differences in depressive symptoms, and when controlling for Attention, such differences no longer evident.
**INTERPERSONAL EFFECTS**				
Austin et al., 2007	Study 1: EQ-i:S, MSCEIT. Study 2: TEIQue-SF	Emotional manipulation	Study 1: *N* = 199 (132 female). *M* = 21.14, *SD* = 3.70. Study 2: *N* = 341 (232 female). *M* = 40.00, *SD* = 19.90	Emotional manipulation correlated positively with Machiavellianism but not significantly with EI. TEI results showed that those high on Machiavellianism struggled most with interpersonal EI (i.e., handling emotions of others), whereas AEI measures indicated they struggled with identifying and understanding own emotions.
Austin et al., 2014	TEIQue-SF	Managing others' emotions	*N* = 369 (246 female). *M* = 18.63, *SD* = 2.03	Although EI correlates negatively with the tendency to worsen others' moods in general, agreeableness moderated this relationship. Low agreeableness produced a much weaker negative relationship, i.e., greater tendency to worsen others' moods in those with high EI.
Bacon et al., 2014	TEIQue-SF	Sensation seeking and delinquency	*N* = 96 (48 female). Males: *M* = 19.83, *SD* = 1.63. Females: *M* = 19.60, *SD* = 1.11.	EI positively related to both sensation seeking and number of delinquent behaviors in females, and did not moderate the relationship between sensation seeking and delinquent behavior as it did for males.
Bacon and Regan, 2016	TEIQue-SF	Manipulative behavior and delinquency	*N* = 252 (125 female). Males: *M* = 20.53, *SD* = 2.66. Females: *M* = 20.33, *SD* = 2.24	Although EI negatively related to general and interpersonal deviancy in males, strongly positively related in females. In females, EI also positively related with use of Machiavellian tactics and morals.
Brackett et al., 2005	MSCEIT	Relationship quality in couples	*N* = 172 (86 female). *M* = 19.7, *SD* = 3.0	EI in one or both partners did not significantly improve relationship quality, although both low EI had more negative outcomes. Couples with both partners having high EI often scored lower on relationship outcomes than those with less collective EI.
Baker et al., 2013	TEIQue-SF	Ability to detect lies	*N* = 116 (83 female, 1 unreported). *M* = 20.1, *SD* = 3.2.	High EI (particularly perceiving/expressing subscales) associated with overconfidence in detecting high-stakes lies, and with greater affective engagement with deceptive targets.
Côté et al., 2011	MSCEIT	Interpersonal deviance	Study 2: *N* = 252 (73% female). *M* = 39.29, *SD* = 10.16.	Emotion regulation knowledge negatively related to interpersonal deviance, but moderated the relationship between Machiavellianism and interpersonal deviance (stronger ERK produced greater interpersonal deviance in individuals with Machiavellian tendencies).
Fix and Fix, 2015	EQ-i	Psychopathy and criminal thinking	*N* = 111 (all male). *M* = 20.58, *SD* = 2.35	Interpersonal Relationships and Stress Management facets significantly predicted psychopathy, and Empathy and Social Responsibility were also significantly positively related.
Grieve and Mahar, 2010	SSEIT	Emotional manipulation	Study 1: *N* = 73 (58 female). *M* = 23.96, *SD* = 10.03. Study 2: *N* = 275 (187 female). *M* = 23.53, *SD* = 8.92	Medium positive correlation (*r* = 0.29) between EI and emotional manipulation, although much lower in females (*r* = 0.07). Higher levels of EI and primary psychopathy predicted greater emotional manipulation in males and females, although in females EI acted as a suppressor variable in psychopathy/manipulation link.
Grieve and Panebianco, 2013	SSEIT	Emotional manipulation	*N* = 243 (155 female). *M* = 34.34, *SD* = 10.27	High EI worked in conjunction with secondary psychopathy in males, and primary and secondary psychopathy in females, to predict higher emotional manipulation. The presence of high EI suppressed variance in other variables and produced stronger prediction of emotional manipulation.
Hyde and Grieve, 2014	SSEIT	Emotional manipulation	*N* = 234 (193 female). *M* = 32.16, *SD* = 13.10	EI a strong predictor in the multivariate model (alongside gender, primary, and secondary psychopathy) of perceived ability to emotionally manipulate others. For females, EI acted as a suppressor variable.
Moeller and Kwantes, 2015	Wong and Law EI Scale	Conflict management behaviors	*N* = 109 (86 female). *M* = 21.32, *SD* = 4.33.	EI moderated the relationship between conflict preferences and behaviors undermining others' esteem, and engaging in confrontational discussion.
Porter et al., 2011	TEIQue-SF	Simulating emotions	*N* = 100 (75 female). *M* = 20.78.	Individuals higher in EI (particularly in perceiving/expressing subscales) were able to produce more convincing deceptive displays of emotion and to continue these displays for longer. They were no better at concealing felt emotions.
Puglia et al., 2005	MSCEIT	EI of offender	*N* = 56 (all male). Sex offenders *M* = 44.42, *SD* = 10.65. Non-sex offending prisoners *M* = 31.72, *SD* = 6.22. Control *M* = 43.37, *SD* = 11.03	Sex offenders scored significantly higher on Perception than non-sex offending prisoners, and overall higher (although not significant) on all branches (Perception, Assimilation, Management) of EI measured than both non-sex offending and control groups.
Tett et al., 2012	MEIA	EI test faking	*N* = 182 (78% female). *M* = 20.40.	Greater faking on self-report EI measure in participants with higher cognitive ability, more opportunity to fake and also on job-relevant traits.
Vidal et al., 2010	MSCEIT	Psychopathy	*N* = 188 (all male). *M* = 19.9, *SD* = 2.7.	No significant relationship between psychopathy and EI (although mildly negative). However, some facets of primary psychopathy (e.g., Fearless Dominance) correlated positively with Facilitating Thoughts facet of EI. High-anxious psychopathy group had significantly lower EI than low-psychopathy group and low-anxious psychopathy group, who had fairly intact EI.

## Intrapersonal effects

Emotionally intelligent individuals should be adept at coping with the demands of everyday life (Bar-On, 2006) with competent affect regulation seen as crucial for psychological wellbeing (Mayer and Salovey, 1997). This skill is supported by lower order EI abilities (perceiving; using emotion to facilitate thinking; understanding emotion), which contribute to a fundamental emotional awareness necessary for adaptive emotion management. It is argued that beliefs about emotional abilities (indicated by TEI) are just as important, given appraisal and reactivity to everyday activities may be partly filtered through these (Petrides et al., 2007). Whilst there is evidence to link EI to better mental and physical health (for reviews see Martins et al., 2010; Resurreccion et al., 2014), research suggests that in some contexts, high levels of EI (particularly emotional awareness; management) may be related to poorer psychological health and adversely impact upon an individual's capacity to deal with emotionally salient situations.

### Psychological (ill) health

Most studies utilized measures of TEI, particularly the Trait Meta-Mood Scale—a multidimensional assessment of mood awareness. For example, attention to emotions was positively associated with greater negative emotional impact (annoyance; dejection) in victims of cyber-bullying (Elipe et al., 2015) and higher levels of symptomatology in patients with borderline personality or anxiety disorders (Lizeretti et al., 2012). Moreover, both studies propose that an imbalance in constituent components of TEI may characterize vulnerability, i.e., an excessive awareness of (negative) emotions coupled with a lack of competency to repair these emotional states resulted in greater psychological discomfort. Extremera and Fernandez-Berrocal (2006) found the same TEI profile predicted mood disorders in young people suggesting this may underpin ruminative thinking known to prolong depressed mood. This appears particularly true in females, where greater attention to emotions accounted entirely for sex differences in depressive symptoms (Thayer et al., 2003).

Explanatory pathways appear less straightforward with regard to AEI. Higher levels of emotional skill have been found to amplify the effects of chronic stressors (socio-economic adversity; daily hassles) on depression, hopelessness and suicidal ideation (Ciarrochi et al., 2002; Davis and Humphrey, 2012b). In Ciarrochi et al.'s study this effect was restricted to skill in emotion perception leading the authors to speculate that a “hyper-awareness” of negative emotion-laden situations may contribute to mental ill health. To corroborate this trend, AEI related to fewer stress symptoms when self-perceived emotional clarity and attention were either uniformly high or low, but was not beneficial for individuals experiencing intense emotions who reported a lack of emotional understanding (Gohm et al., 2005). It may also be the case that TEI and AEI work in tandem to reinforce these effects; high levels of emotional skill yet low emotional self-efficacy, result in higher levels of depression in adolescent and adults (Davis and Humphrey, 2014; Salguero et al., 2015).

### Stress (over) reactivity

Possible mechanisms of heightened emotional sensitivity may lie in the relationship between EI and stress reactivity. Although those with higher level of TEI show minimal mood deterioration, heart rate variation, and cortisol release in response to stressors (Mikolajczak et al., 2007; Laborde et al., 2011), AEI appears to be positively related to greater cortisol reactivity and slower recovery from situational (lab-induced) social stress—particularly in those with high ability to perceive threat-related emotions (Bechtoldt and Schneider, 2016). Enhanced reactivity has also been documented via subjective reports of mood change—those with high AEI reported an increase in post-task distress, despite reduced pre-task levels, compared to those lower in AEI (Matthews et al., 2006). Mood induction studies have shown that there are also instances where TEI may contribute to greater self-reported mood deterioration (less positive and greater negative affect) following exposure to negative mood manipulation (Petrides and Furnham, 2003; Fernández-Berrocal and Extremera, 2006, study 2; Sevdalis et al., 2007, study 1).

Detrimental effects have been similarly demonstrated in stressful or high-stakes applied settings. For male (not female) students, high levels of emotion perception and management related to poorer delivery of a timed speech to an audience, possibly due to a hyper-awareness of emotional reactivity to a contentious topic (sexual harassment) and the need to control this response (Lyons and Schneider, 2005). University students with uniformly high or average TEI profiles appear more likely to graduate compared to those with an “uneven” pattern of skills; low-average interpersonal/intrapersonal skills and adaptability coupled with high stress management yielded higher rates of degree non-completion (Keefer et al., 2012). In a similar vein, high TEI nursing students fared no better than those with very low levels in terms of experiencing post-traumatic growth following exposure to childhood adversity—average levels of EI were most beneficial (Li et al., 2015). Moreover, nurses appear to decrease caring behaviors toward their patients, despite high empathy levels, if they also have high TEI self-control (Rego et al., 2010). It may be the case that these “over-controlled” individuals are unwilling to translate knowledge (empathic concern) into practice for reasons of personal safeguarding (i.e., compassion fatigue). Recent research hints at the complexity involved in this relationship (Zeidner et al., 2013) and clearly further research is required in applied settings to verify the adaptive nature of EI. Nevertheless, research alludes to the notion of an optimal balance between emotional sensitivity and management for successful adaptation.

## Interpersonal effects

High TEI typically reflects extroverted (happy; optimistic), agreeable (low assertiveness), conscientious (self-motivated), confident, and emotionally stable individuals (Petrides, 2009). High levels of AEI reflect superior emotional knowledge, awareness, and regulatory ability. Both types of EI therefore subsume tools and qualities that appear useful for successful navigation of social exchanges. Whilst high EI has been associated with more satisfying relationships and pro-social behaviors (e.g., Lopes et al., 2004; Frederickson et al., 2012), a growing pool of studies suggest that EI may be used as a tool for manipulative ends, with high scorers skilled in emotional distortion and antisocial behaviors. This may result in adaptive accomplishment of personal self-serving goals but may also thwart the development of satisfying interpersonal relationships. For instance, within a romantic relationship, one partner having high TEI appears to improve relationship quality, yet two high scorers relates to poorer outcomes—possibly due to competitive over-management of the relationship, or an acute awareness of emotional idiosyncrasies (Brackett et al., 2005). This section reviews evidence linking high EI to emotional manipulation and antisocial behaviors (e.g., aggression, deviance).

### Emotional manipulation and deception

Direct associations between A/TEI and emotional manipulation are inconsistent—either moderately positive or absent (Austin et al., 2007; Grieve and Mahar, 2010; Grieve and Panebianco, 2013). So too are correlations between facets of psychopathy (sensation-seeking, fearlessness, aggression), AEI and TEI—with some suggesting that skill in using emotion, stress management, and interpersonal interaction are predictive of psychopathy (Vidal et al., 2010; Fix and Fix, 2015) whilst others find no relationship (Lishner et al., 2011). There is, however, converging evidence to suggest that EI operates indirectly, in conjunction with allied traits and skills, to predict manipulative behaviors. Grieve and Panebianco (2013) found that males with higher levels of TEI, social information processing, indirect aggression, and self-serving cognitive distortions were more likely to exploit others. This pattern varied according to sex, with age, primary psychopathic traits, and social awareness being additionally important predictors of manipulation in females. Grieve and Mahar (2010) found similar multi-facetted sex differences—whilst primary psychopathy and high levels of TEI conjointly predicted emotional manipulation for both males and females, ethical reasoning and secondary psychopathy were important correlates for females only. The predictive effect of high levels of psychopathic traits/TEI on manipulative behavior was replicated again more recently (Hyde and Grieve, 2014). Consequently, the way in which EI is deployed (i.e., for better or worse) appears contingent upon other underlying pre-dispositions and competencies of the individual.

This manipulative ability may, in part, be underscored by a natural capacity deceive. Analysis of self-report data showed that those with high TEI and cognitive ability were more likely to fake on high stakes measures (Tett et al., 2012). Objectively, Porter et al. (2011) found that high TEI emotionality (perceiving and expressing emotion) was related to an increased ability to simulate deceptive emotions and to persist in these displays for longer. Conversely, those high in TEI wellbeing (happiness; optimism) were no better at masking their true felt emotions, suggesting an association between TEI and a tendency toward emotional openness. This perhaps explains why high TEI (emotionality) is associated with a propensity for gullibility and overestimation of others' honesty (Baker et al., 2013).

### Antisocial behavior

Austin et al. (2014) found trait agreeableness mediated the relationship between TEI and use of non-prosocial behaviors (e.g., worsening others' moods), such that at low levels of agreeableness, those high in TEI were prone to such behavior. AEI may also moderate antisocial behaviors. Côté et al. (2011) found that whilst skill in emotion management was not directly related to interpersonal deviance, it served to intensify the relationship between Machiavellianism and deviance, acting as a useful tool for those with a propensity to harm. Similarly TEI emotion management and understanding increase the tendency to engage in confrontation in those who view this as an effective negotiation strategy (Moeller and Kwantes, 2015). This self-serving element of EI could therefore present as selfish and aggressive behavior toward others. Sex differences are also evident with high TEI reducing Machiavellian tactics, moral thinking, and delinquency in males, yet promoting all of these aspects in females (Bacon and Regan, 2016) who may rely more on emotive, relational forms of aggression, and manipulation (Bacon et al., 2014).

## Discussion

**Figure 1:**
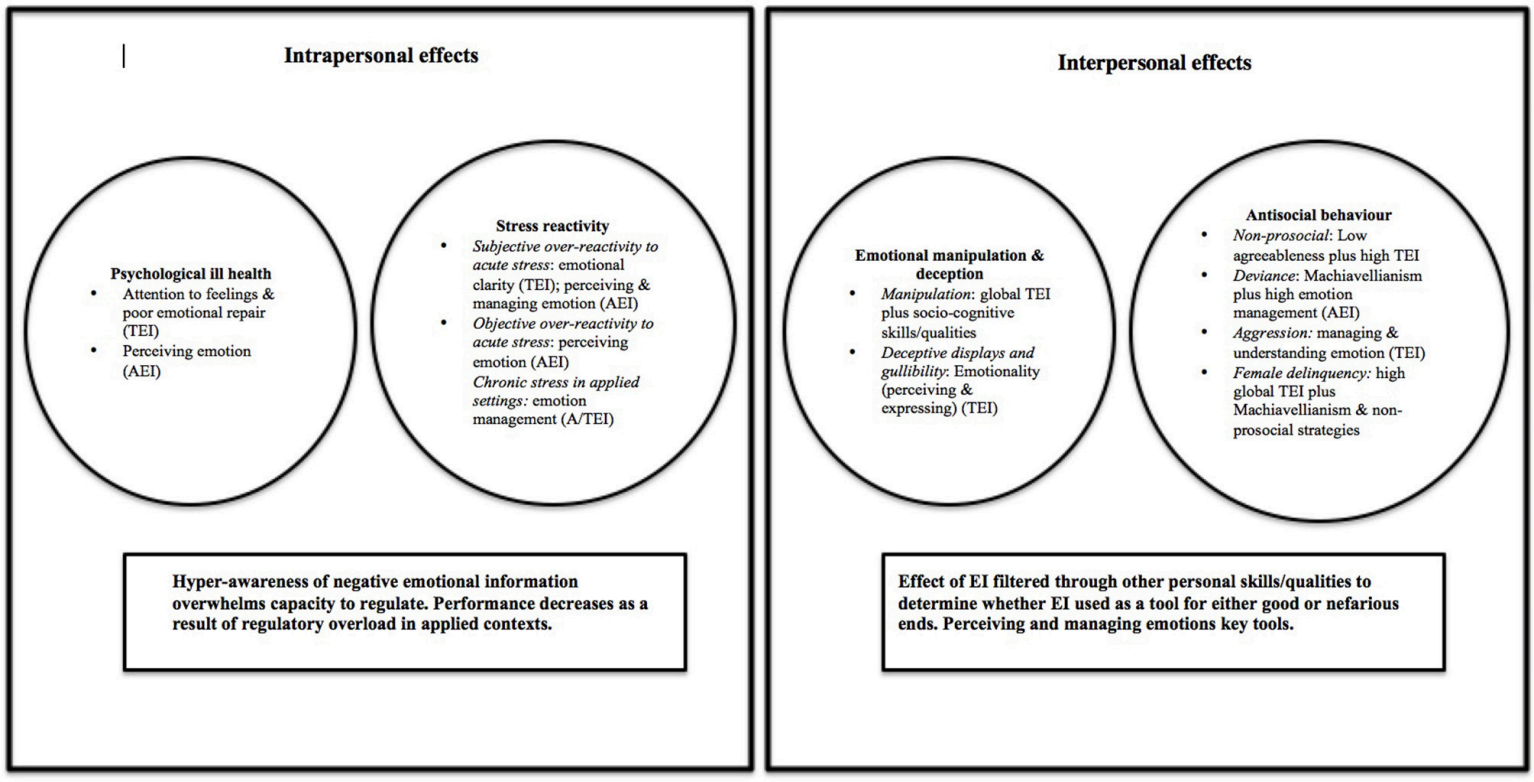
**Summary of emergent “dark” EI themes**.

This review finds embryonic support for the notion of “dark” EI. There are contexts in which it is not universally beneficial to have high levels of EI—whether trait or ability. This can translate into negative effects for self (psychological ill-health; stress reactivity) and/or others (manipulative, antisocial behaviors; see thematic overview: Figure 1). Whilst prevailing EI literature discusses positive effects of possessing high EI in intrapersonal and interpersonal domains (e.g., Joseph and Newman, 2010; Martins et al., 2010), this review synthesizes a growing number of anomalous findings that do not concur with this perspective. A number of key themes emerged concerning when, why and how might EI be deleterious or harmful.

Dark effects spanned multiple environmental contexts (academic; health; occupational) across intrapersonal and interpersonal functioning. There was a trend toward individual-level contextual effects, with sex differences evident in psychological health, stress reactivity, emotional manipulation, and delinquency. Across both A/TEI domains, emotion management, and perception were most often implicated in negative outcomes. However, profiles of *within-person vulnerability* exist, particularly for intrapersonal outcomes, suggesting a balance between EI facets is optimal for adaptation. For instance, high emotional awareness, low regulation, and/or understanding relates to sub-clinical symptomatology (e.g., Gohm et al., 2005; Extremera and Fernandez-Berrocal, 2006). It is important that EI researchers move away from traditional bivariate methods to use person-centered analytical techniques (e.g., latent profile analysis) to allow further scrutiny of multi-facetted EI in action (Keefer et al., 2012). A profile of *cross-construct within-person vulnerability* may also exist. Whilst few studies have examined this possibility by including multiple measures of EI, having high levels of emotional skill (AEI) and low emotional self-confidence (TEI) to translate skills into practice, appears deleterious (Davis and Humphrey, 2014). Hence there could be an optimal level at which each type of EI is useful before effects become negative. Finally, EI operated indirectly to modify or jointly predict dark effects via underlying socio-cognitive skills/qualities (e.g., agreeableness; cognitive ability) and environmental contexts (e.g., stress). There is therefore an urgent need to study “EI in action” by modeling moderating and mediating effects to better understand how and when EI is deployed. For instance, findings suggest that deleterious intrapersonal effects (e.g., poor mental health) could stem from hypersensitivity to emotional information, which manifests in particular stressful situations (e.g., socio-economic adversity; public speaking). Researchers must examine why this arises; for instance, do those with high levels of emotional skill show differing patterns of attentional bias for threatening emotional information when under stress? Does this apply to all types of stress or is this selective? How does this relate to different intra and interpersonal outcomes in particular groups (e.g., clinical, academic, occupational)? It is possible that a hyperawareness of emotional cues/consequences conveys interpersonal advantages in competitive job roles where climbing the social hierarchy is rewarded (contingent perhaps on possessing other key traits/abilities e.g., high cognitive ability).

The relatively small pool of literature, disparate range of EI tools utilized and unsophisticated analytical methods make it difficult to identify precisely what optimal EI might be and in which context this might arise. So far, average EI levels appear most beneficial for adaptation (e.g., Li et al., 2015), and individuals with low to average levels gain the most from training interventions (Keefer et al., 2012). Methodological techniques for the study of non-monotonic effects should now be routinely applied to EI research to aid this endeavor (for examples see Davis and Humphrey, 2012a; Li et al., 2015). Researchers must examine EI in context (multivariable models; curvilinear trends; indirect pathways; measure of “impact”) and control for the overlapping influence of allied personality traits (Veselka et al., 2012). This is important for construct coherency e.g., impulse control/self-regulatory behavior is indicated by measures of emotion manipulation, “Big Five,” and EI. Additionally, the lack of longitudinal research makes it challenging to determine how deleterious outcomes might present developmentally, whether they are long lasting and ultimately, whether training EI is beneficial.

## Author contributions

Substantial contributions to the conception or design of the work (SD); or the acquisition, analysis, or interpretation of data for the work (SD/RN); Drafting the work or revising it critically for important intellectual content (SD/RN); Final approval of the version to be published (SD/RN). Agreement to be accountable for all aspects of the work in ensuring that questions related to the accuracy or integrity of any part of the work are appropriately investigated and resolved (SD/RN).

### Conflict of interest statement

The authors declare that the research was conducted in the absence of any commercial or financial relationships that could be construed as a potential conflict of interest.
